# Rosmarinic Acid Elicits Calcium-Dependent and Sucrose-Sensitive Eryptosis and Hemolysis through p38 MAPK, CK1α, and PKC

**DOI:** 10.3390/molecules28248053

**Published:** 2023-12-12

**Authors:** Sumiah A. Alghareeb, Mohammad A. Alfhili, Jawaher Alsughayyir

**Affiliations:** Department of Clinical Laboratory Sciences, College of Applied Medical Sciences, King Saud University, Riyadh 12372, Saudi Arabia; 442204700@student.ksu.edu.sa (S.A.A.); malfeehily@ksu.edu.sa (M.A.A.)

**Keywords:** eryptosis, hemolysis, rosmarinic acid, chemotherapy

## Abstract

Background: Rosmarinic acid (RA) possesses promising anticancer potential, but further development of chemotherapeutic agents is hindered by their toxicity to off-target tissue. In particular, chemotherapy-related anemia is a major obstacle in cancer therapy, which may be aggravated by hemolysis and eryptosis. This work presents a toxicity assessment of RA in human RBCs and explores associated molecular mechanisms. Methods: RBCs isolated from healthy donors were treated with anticancer concentrations of RA (10–800 μM) for 24 h at 37 °C, and hemolysis and related markers were photometrically measured. Flow cytometry was used to detect canonical markers of eryptosis, including phosphatidylserine (PS) exposure by annexin-V-FITC, intracellular Ca^2+^ by Fluo4/AM, cell size by FSC, and oxidative stress by H_2_DCFDA. Ions and pH were assessed by an ion-selective electrode, while B_12_ was detected by chemiluminescence. Results: RA elicited concentration-dependent hemolysis with AST and LDH release but rescued the cells from hypotonic lysis at sub-hemolytic concentrations. RA also significantly increased annexin-V-positive cells, which was ameliorated by extracellular Ca^2+^ removal and isosmotic sucrose. Furthermore, a significant increase in Fluo4-positive cells and B_12_ content and a decrease in FSC and extracellular pH with KCl efflux were noted upon RA treatment. Hemolysis was augmented by blocking KCl efflux and was blunted by ATP, SB203580, staurosporin, D4476, isosmotic urea, and PEG 8000. Conclusions: RA stimulates Ca^2+^-dependent and sucrose-sensitive hemolysis and eryptosis characterized by PS exposure, Ca^2+^ accumulation, loss of ionic regulation, and cell shrinkage. These toxic effects were mediated through energy deprivation, p38 MAPK, protein kinase C, and casein kinase 1α.

## 1. Introduction

Chemotherapy-associated anemia (CAA) is a major obstacle in cancer treatment, with a prevalence of at least 75% in patients undergoing therapy [1]. Although complex, the underlying mechanisms of CAA involve decreased RBC production due to myelosuppression and the premature death of circulating cells by either hemolysis or eryptosis [2]. Suicidal RBC death is characterized by phospholipid scrambling of the cell membrane, loss of ionic homeostasis, reduced cellular volume, oxidative stress, ceramide accumulation, and metabolic deprivation. Hemolysis and eryptosis are regulated by caspase, p38 MAPK, casein kinase 1α (CK1α), protein kinase C (PKC), Rac1 GTPase, and JAK3, among other kinases. Loss of membrane asymmetry results in phosphatidylserine (PS) exposure to the outer membrane leaflet, which serves as a binding site for recognition and engulfment by phagocytes, which predisposes to anemia [3]. 

**Figure 1:**
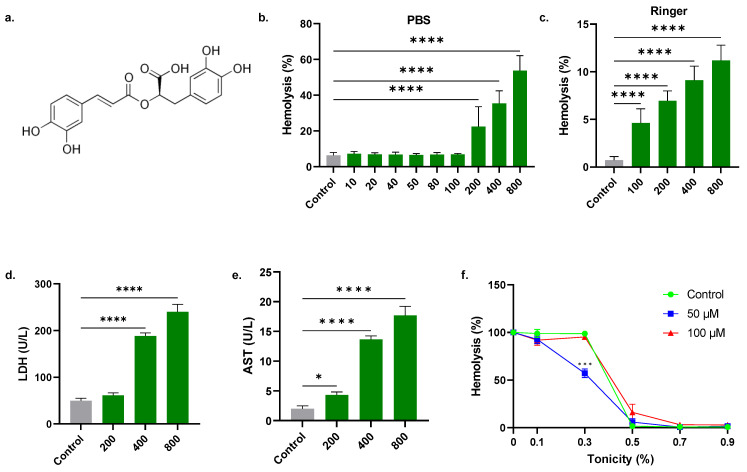
RA stimulates concentration-dependent hemolysis. Molecular structure of RA (**a**), hemolytic rate of RA in PBS (**b**), and in (**c**) standard Ringer solution. Release of (**d**) LDH and (**e**) AST in the supernatant. (**f**) Antihemolytic effect of RA in hypotonic conditions. * (*p* < 0.05), *** (*p* < 0.001), and **** (*p* < 0.0001).

Rosmarinic acid (RA) is a polyphenolic ester of caffeic acid (Figure 1a) isolated from various plants, most notably rosemary (*Rosmarinus officinalis*), sage (*Salvia officinalis*), basil (*Ocimum tenuiflorum*), oregano (*Origanum vulgare*), and lemon balm (*Melissa officinalis*) [4]. RA exhibits anti-inflammatory, antimicrobial, anticancer, immunomodulatory, and antioxidant properties [5,6]. Importantly, RA triggers apoptosis in leukemia, breast, gastric, hepatocellular, colorectal, glioma [7], melanoma [8], prostate [9], and lung cancer cells [10,11,12]. Mechanisms involved in RA-induced apoptosis include targeting the cell cycle and DNA damage [13], histone deacetylases [9], mitochondrial Bcl-2 and Bax, caspases, PI3K/Akt/NF-κB [7], and ADAM17/EGFR/AKT/GSK3β [14].

Although RA has promising anticancer potential, no study has thus far examined its toxicity to human RBCs. Our aim is thus to investigate the interaction of RA with RBCs and identify the underlying molecular mechanisms.

## 2. Results

### 2.1. A Stimulates Concentration-Dependent Hemolysis

Cells treated with 10 to 800 μM of RA in PBS exhibited concentration-dependent hemolysis (Figure 1b). Compared to control values of 6.39 ± 1.59%, statistical significance was achieved at 200 μM (22.50 ± 11.09%), 400 μM (35.43 ± 7.02%), and 800 μM (53.76 ± 8.31%). Similarly, in Ringer solution (Figure 1c), control values (0.71 ± 0.39%) significantly increased to 4.61 ± 1.50% (100 μM), 6.96 ± 1.03% (200 μM), 9.12 ± 1.48% (400 μM), and 11.18 ± 1.62% (800 μM). Moreover, hemolysis was accompanied by a substantial increase in LDH release from 49.33 ± 5.51 U/L to 188.0 ± 7.00 U/L and 240.0 ± 16.09 U/L at 400 and 800 μM of RA, respectively (Figure 1d). In addition, a notable increase in AST was observed from 2.00 ± 0.50 U/L to 4.33 ± 0.49 U/L (200 μM), 13.67 ± 0.58 U/L (400 μM), and 17.67 ± 1.53 U/L (800 μM), as illustrated in Figure 1e. It is also revealed that 50 µM of RA enhanced the osmotic resistance of RBCs (Figure 1f). This is evident as RA significantly suppressed hypotonic lysis (0.3% NaCl) in comparison to the negative control (98.77 ± 1.64% vs. 57.23 ± 4.48%).

### 2.2. RA Triggers Eryptosis

**Figure 2:**
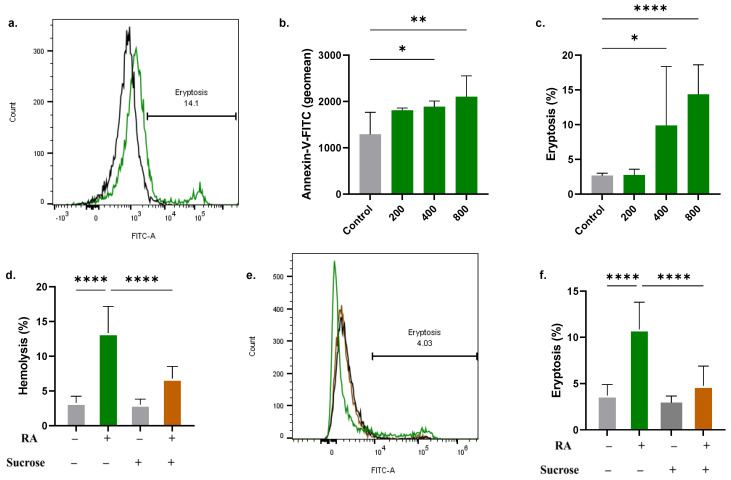
RA triggers eryptosis. (**a**) Representative histograms of annexin−V−FITC fluorescence in control (black line) and experimental cells (green line, 800 μM). (**b**) Geomean of annexin−V−FITC fluorescence (**c**) and percentage of eryptotic cells. (**d**) Effect of isosmotic sucrose on RA−induced hemolysis (800 μM). (**e**) Representative histograms of annexin−V−FITC fluorescence in the absence (green line) and presence (brown line) of sucrose. (**f**) percentage of eryptotic cells in the presence and absence of sucrose. * (*p* < 0.05), ** (*p* < 0.01), and **** (*p* < 0.0001).

As depicted in Figure 2b, cells treated with RA exhibited a rise in the geometric mean of annexin-V-FITC fluorescence. Control values of 1290 ± 474.9 arbitrary units (a.u.) were significantly elevated to 1886 ± 122.6 a.u. (400 µM) and 2102 ± 450.5 a.u. (800 µM). The percentage of cells with PS exposure was also increased from 2.66 ± 0.34% to 9.86 ± 8.50% (400 µM) and 14.33 ± 4.28% (800 µM), as evident from Figure 2c. Furthermore, the addition of 250 mM of sucrose had a notable protective effect on the cells against RA-induced hemolysis (13.34 ± 3.81% to 6.79 ± 1.75%, Figure 2d). A significant decrease in annexin V-binding cells was also noted in the presence of sucrose (10.86 ± 2.92% to 4.77 ± 2.12%, Figure 2f). 

### 2.3. RA Increases Cytosolic Ca^2+^

**Figure 3:**
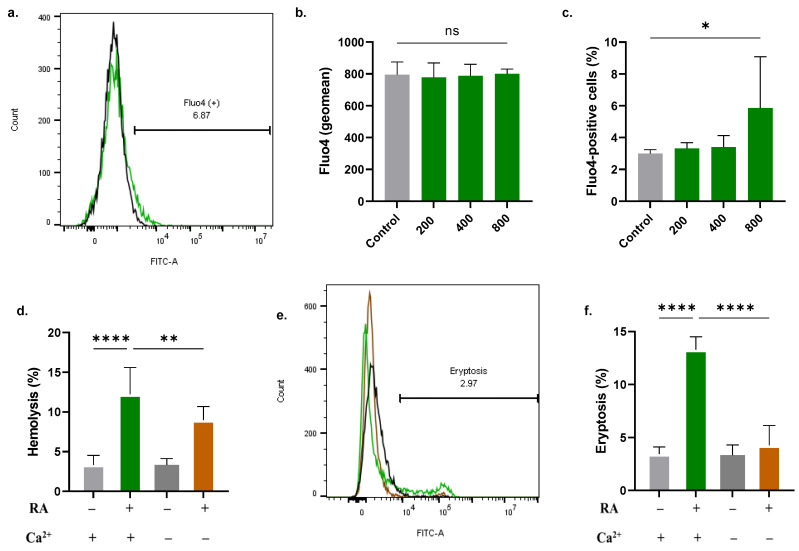
RA increases cytosolic Ca^2+^ levels. (**a**) Representative histograms of Fluo4 fluorescence in control (black line) and experimental (green line, 800 μM) cells. (**b**) Geomean of Fluo4 fluorescence and (**c**) percentage of cells with Ca^2+^ accumulation. (**d**) Effect of extracellular Ca^2+^ elimination on hemolysis. (**e**) Representative histograms of annexin-V-FITC fluorescence with (green line) and without (brown line) extracellular Ca^2+^. (**f**) Effect of extracellular Ca^2+^ elimination on eryptosis. No significance is indicated by ns, while * (*p* < 0.05), ** (*p* < 0.01), and **** (*p* < 0.0001).

The percentage of RBCs with elevated Ca^2+^ levels was significantly greater in cells exposed to 800 µM of RA (3.00 ± 0.24% to 5.86 ± 3.22%, Figure 3c). Also, Figure 3d illustrates a statistically significant reduction in hemolysis when extracellular Ca^2+^ is removed (12.22 ± 3.36% to 8.98 ± 1.72%). Likewise, eryptosis was significantly reduced in the absence of Ca^2+^ (13.29 ± 1.22% to 4.25 ± 1.88%) (Figure 3f).

### 2.4. RA Leads to Cell Shrinkage

**Figure 4:**
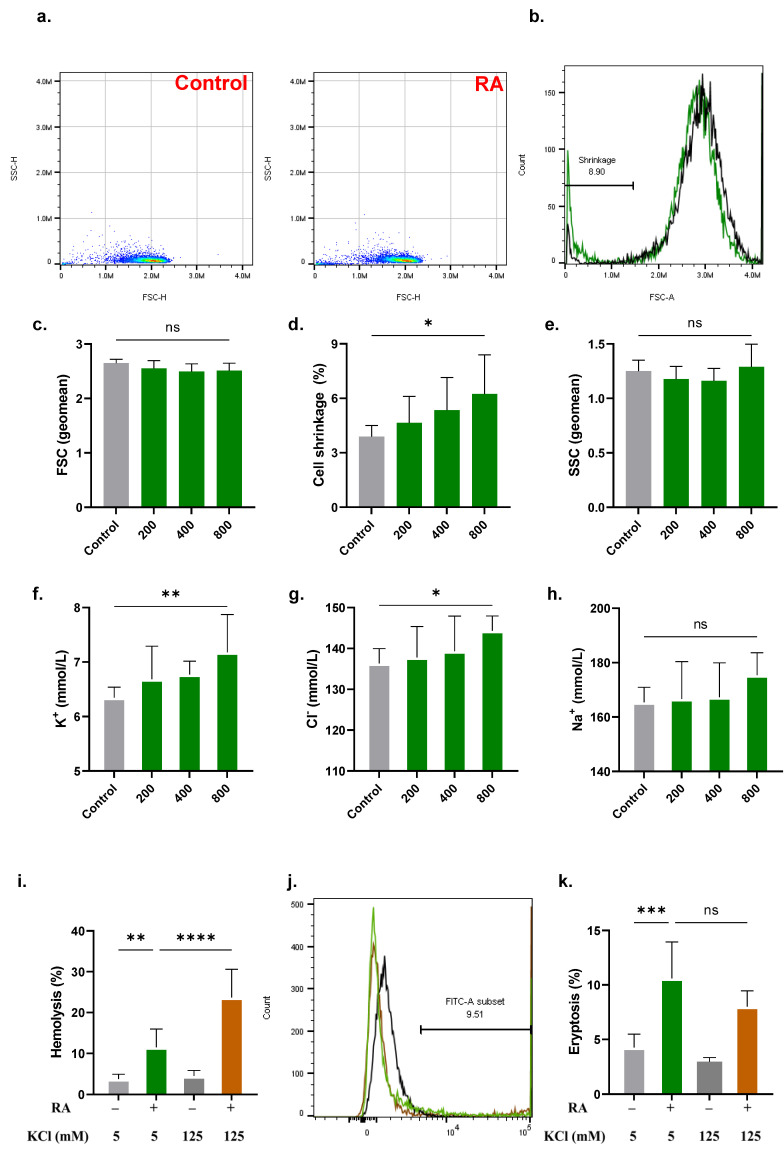
RA leads to cell shrinkage. (**a**) Representative dot plots of SSC and FSC signals. (**b**) Representative histograms of the FSC distribution of control (black line) and experimental (green line, 800 μM) cells. (**c**) Geomean of FSC, (**d**) percentage of shrunk cells, and (**e**) geomean of SSC. Leakage of (**f**) K^+^, (**g**) Cl^−^, and (**h**) Na^+^. (**i**) Effect of 125 mM of KCl on RA-induced hemolysis. (**j**) Representative histograms of annexin-V-FITC fluorescence in 5 mM of KCl (green line) and 125 mM of KCl (brown line). (**k**) Effect of 125 mM of KCl on RA-induced eryptosis. No significance is indicated by ns, while * (*p* < 0.05), ** (*p* < 0.01), *** (*p* < 0.001), and **** (*p* < 0.0001).

A significant increase in the percentage of cells that underwent shrinkage was observed at 800 μM of RA (3.89 ± 0.61% to 6.24 ± 2.16%), as shown in Figure 4d. Accordingly, at the same concentration, efflux of K^+^ increased from 6.35 ± 0.19 mmol/L to 7.18 ± 0.69 mmol/L (Figure 4f), and that of Cl^−^ increased from 136.3 ± 3.65 mmol/L to 144.3 ± 3.65 mmol/L (Figure 4g). Interestingly, the hemolytic effect of RA was significantly increased in the presence of extracellular 125 mM of KCl when compared to standard Ringer solution (11.53 ± 4.41% to 23.69 ± 6.95%, Figure 4i), while eryptosis was not appreciably affected (Figure 4k).

### 2.5. RA Causes of Morphological Alterations

**Figure 5:**
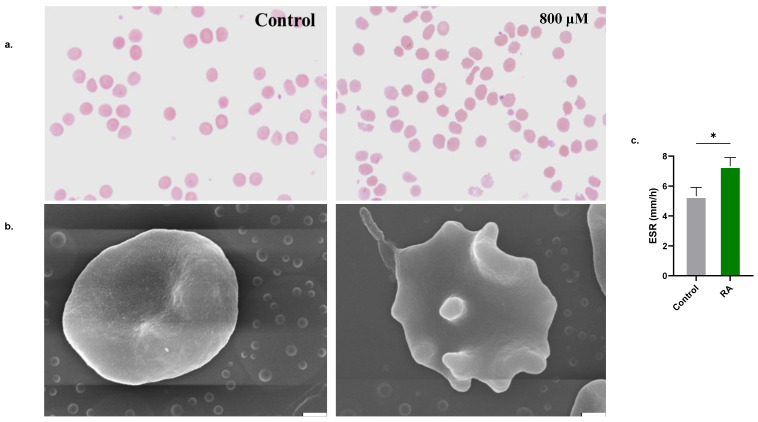
Effect of RA on erythrocyte morphology. (**a**) Giemsa-stained, bright-field images, (**b**) SEM micrographs (×10,000; scale bar: 1 μm), and (**c**) ESR of control and experimental RBCs (800 μM). * (*p* < 0.05).

Treatment of cells with 800 μM of RA resulted in the formation of schistocytes and acanthocytes, as revealed by Giemsa staining (Figure 5a) and SEM analysis (Figure 5b). The ESR of treated cells was also significantly elevated (5.33 ± 0.58 mm/h to 7.33 ± 0.58 mm/h, Figure 5c).

### 2.6. RA Increases Extracellular Acidity and Traps Intracellular B_12_

**Figure 6:**
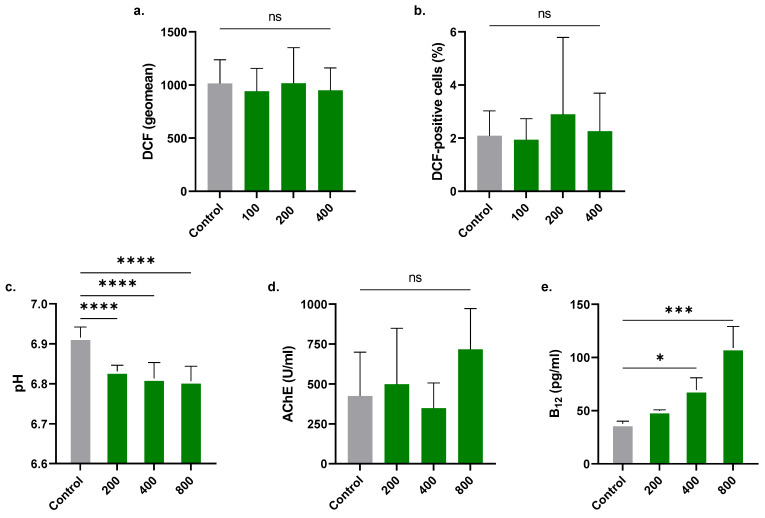
Effect of RA on extracellular acidity and B12. (**a**) Geomean of DCF fluorescence and (**b**) the percentage of oxidized cells. (**c**) Effect of RA on extracellular pH and intracellular (**d**) AChE activity and (**e**) vitamin B12. No significance is indicated by ns, while * (*p* < 0.05), *** (*p* < 0.001), and **** (*p* < 0.0001).

As seen in Figure 6a,b, RA did not result in a significant elevation in oxidative stress. Moreover, as illustrated in Figure 6c, a substantial decrease in extracellular pH was observed at 200 μM (6.83 ± 0.015), 400 μM (6.81 ± 0.040), and 800 μM (6.80 ± 0.037) compared to the control (6.92 ± 0.04). No appreciable effect on AChE activity upon exposure of cells to 200, 400, or 800 µM of RA was noted (Figure 6d). RA also caused a significant increase in intracellular B_12_ from 37.67 ± 2.52 pg/mL to 69.41 ± 11.61 pg/mL (400 µM) and 109.0 ± 20.09 pg/mL (800 µM), as shown in Figure 6e.

### 2.7. Hemolysis Triggered by RA Is Mediated via Several Signaling Pathways

**Figure 7:**
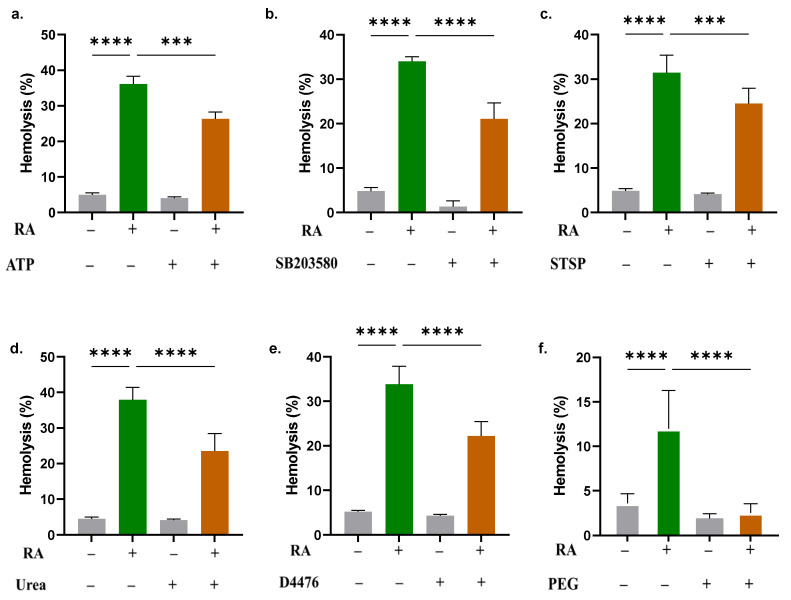
RA-induced cell death is mediated through several pathways. Effect of (**a**) ATP (500 µM), (**b**) SB203580 (100 µM), (**c**) STSP (1 µM), (**d**) urea (300 mM), (**e**) D4476 (20 µM), and (**f**) PEG 8000 (10% *w*/*v*) on the hemolytic activity of RA. All assays were carried out in PBS in the presence of 400 μM of RA, except for PEG, which was in Ringer buffer and against 800 μM of RA. *** (*p* < 0.001) and **** (*p* < 0.0001).

The hemolytic effects caused by RA (400 µM) are mitigated by the administration of ATP (36.10 ± 2.20% to 26.32 ± 1.89%, Figure 7a), SB203580 (33.99 ± 1.07% to 21.07 ± 3.62%, Figure 7b), STSP (31.46 ± 3.94% to 24.49 ± 3.47%, Figure 7c), urea (37.90 ± 3.49% to 23.53 ± 4.90%, Figure 7d), D4476 (33.81 ± 4.06% to 22.20 ± 3.26%, Figure 7e), and PEG 8000 at 10% *w*/*v* (11.98 ± 4.32% to 2.52 ± 1.04%, Figure 7f).

### 2.8. RA Is Cytotoxic to Peripheral Blood Cells

**Figure 8:**
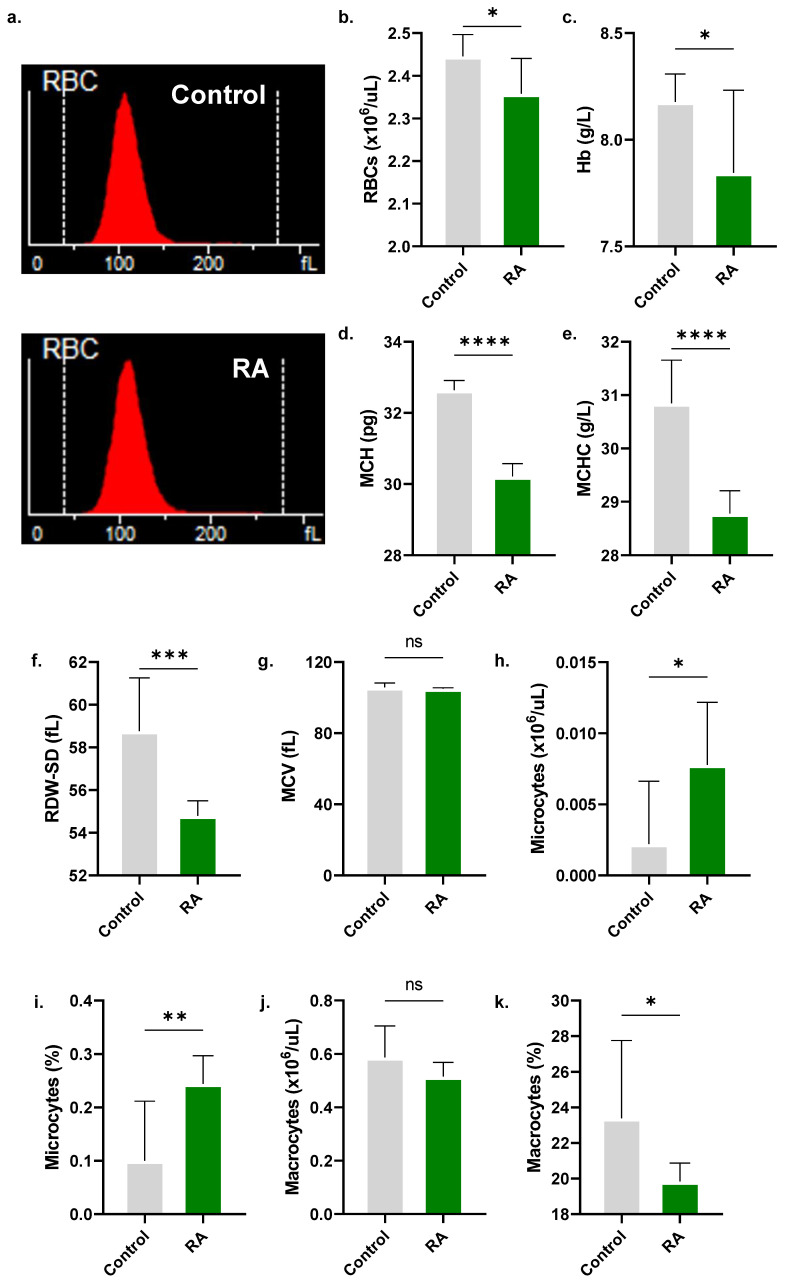
Effect of RA on RBC indices. (**a**) Representative histograms of RBC count and size in control and experimental whole blood. (**b**) RBC count. (**c**) Hb. (**d**) MCH. (**e**) MCHC. (**f**) RDW-SD. (**g**) MCV. (**h**) Microcyte count and (**i**) percentage. (**j**) Macrocyte count and (**k**) percentage. No significance is indicated by ns, while * (*p* < 0.05), ** (*p* < 0.01), *** (*p* < 0.001), and **** (*p* < 0.0001).

**Figure 9:**
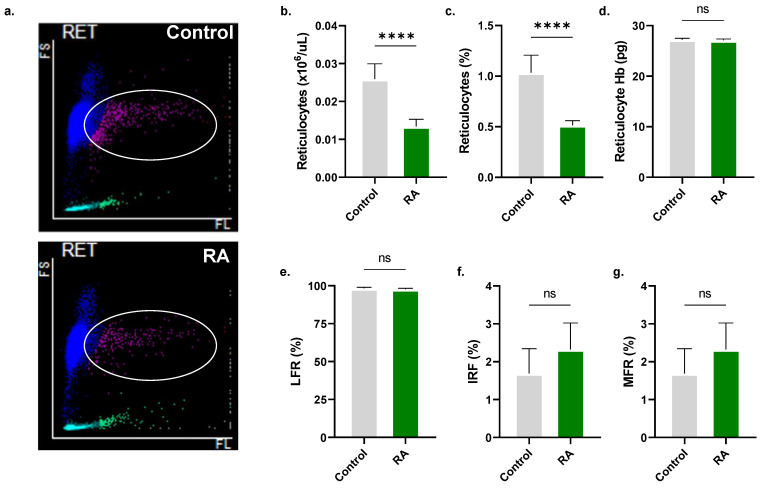
RA is cytotoxic to reticulocytes. (**a**) Representative scattergrams depicting FSC and fluorescence intensity in control and experimental whole blood. (**b**) Reticulocyte count, (**c**) percentage, and (**d**) Hb. (**e**) low-fluorescence reticulocytes (LFR), (**f**) immature reticulocyte fraction (IRF), and (**g**) medium-fluorescence reticulocytes (MFR). No significance is indicated by ns, while **** (*p* < 0.0001).

In whole blood, significant reductions in RBC count (2.45 ± 0.05 × 10^6^/μL to 2.36 ± 0.08 × 10^6^/μL, Figure 8b) and other RBC indices were observed upon treatment with 800 µM RA, including Hb (7.84 ± 0.13 g/L to 8.18 ± 0.39 g/L, Figure 8c), MCH (32.64 ± 0.27 pg to 30.21 ± 0.36 pg, Figure 8d), MCHC (30.84 ± 0.81 g/L to 28.78 ± 0.43 g/L, Figure 8e), and RDW-SD (58.77 ± 2.49 fL to 54.80 ± 0.70 fL, Figure 8f). Moreover, RA significantly increased the proportion of microcytes (0.10 ± 0.11% to 0.24 ± 0.05%, Figure 8i) and decreased that of macrocytes (23.40 ± 4.36% to 19.83 ± 1.05%, Figure 8k). A significant reduction in reticulocytes from 0.03 ± 0.003 × 10^6^/μL to 0.01 ± 0.002 × 10^6^/μL (Figure 9b) and reticulocyte percentage from 1.03 ± 0.17% to 0.51 ± 0.04% (Figure 9c) was also noted.

**Figure 10:**
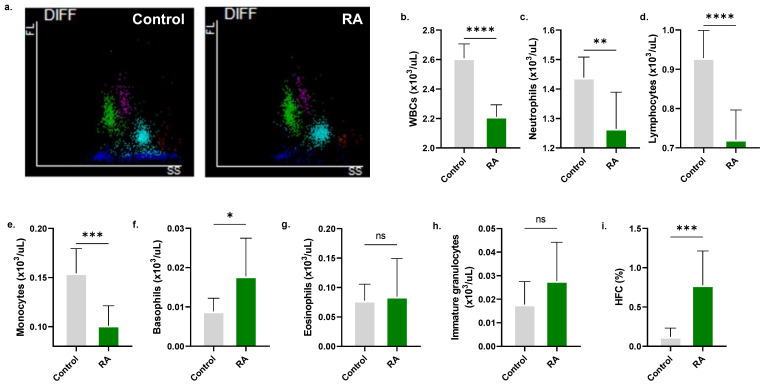
Toxicity of RA to leukocytes. (**a**) Representative scattergrams depict fluorescence intensity and SSC in control and experimental whole blood. (**b**) WBC count. (**c**) neutrophil, (**d**) lymphocyte, (**e**) monocyte, (**f**) basophil, (**g**) eosinophil, and (**h**) immature granulocyte viability. (**i**) high-fluorescent cell (HFC) fraction. No significance is indicated by ns, while * (*p* < 0.05), ** (*p* < 0.01), *** (*p* < 0.001), and **** (*p* < 0.0001).

Similarly, it was observed that RA significantly reduced the viability of leukocytes (2.61 ± 0.09 × 10^3^/μL to 2.21 ± 0.081 × 10^3^/μL, Figure 10b) with specific reductions in neutrophils, lymphocytes, and monocytes, as seen by the decrease in viable cells from 1.44 ±.070 × 10^3^/μL to 1.27 ± 0.12 × 10^3^/μL (Figure 10c), 0.93 ± 0.07 × 10^3^/μL to 0.72 ± 0.08 × 10^3^/μL (Figure 10d), and 0.15 ± 0.03 × 10^3^/μL to 0.10 ± 0.02 × 10^3^/μL (Figure 10e), respectively. Also, the percentage of high-fluorescent cells (HFC), signifying atypical leukocytes, was significantly elevated (0.12 ± 0.11% to 0.78 ± 0.44%, Figure 10i).

**Figure 11:**
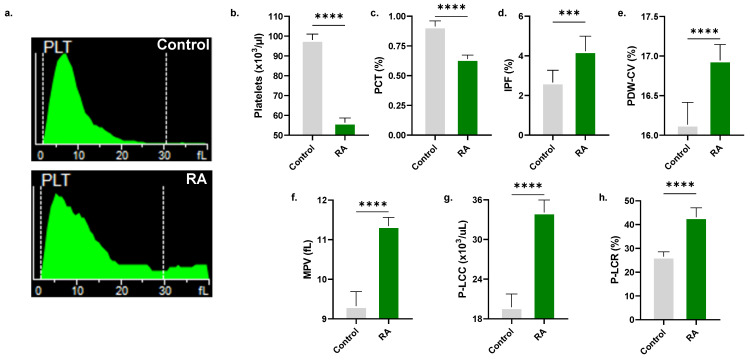
RA exhibits antiplatelet activity. (**a**) Representative histograms of platelet count and size. (**b**) Platelet count. (**c**) Plateletcrit (PCT). (**d**) Immature platelet fraction (IPF). (**e**) Coefficient of variation in platelet distribution width (PDW-CV). (**f**) Mean platelet volume (MPV). (**g**) Platelet-large cell count (P-LCC) and (**h**) platelet-large cell ratio (P-LCR). *** (*p* < 0.001) and **** (*p* < 0.0001).

RA also demonstrated specific cytotoxicity toward platelets (98.11 ± 2.89 × 10^3^/μL to 56.33 ± 2.35 × 10^3^/μL, as depicted in Figure 11b), along with diminished plateletcrit (0.91 ± 0.05% to 0.64 ± 0.03%, Figure 11c). Significant elevations were seen in immature platelet fraction (2.66 ± 0.62% to 4.23 ± 0.76%, Figure 11d), PDW-CV (16.13 ± 0.28% to 16.94 ± 0.20%, Figure 11e), MPV (9.32 ± 0.37 fL to 11.34 ± 0.22 fL, Figure 11f), and number (19.76 ± 2.00 × 10^3^/μL to 34.10 ± 1.89 × 10^3^/μL, Figure 11g) and proportion (26.56 ± 2.01% to 43.11 ± 3.95%, Figure 11h) of large platelets.

## 3. Materials and Methods

### 3.1. Chemicals and Reagents

All chemicals were of analytical grade and were obtained from Solarbio Life Science (Beijing, China). RA (CAS #20283-92-5) was purchased as a pure compound extracted and purified from the *Rosmarinus officinalis* shrub (purity ≥ 98%). To prepare a 50 mM stock solution of RA, 10 mg were dissolved in 555 μL of DMSO, and aliquots were stored at −80 °C. Standard Ringer solution was composed of 125 mM NaCl, 5 mM KCl, 1 mM MgSO_4_, 32 mM HEPES, 5 mM glucose, and 1 mM CaCl_2_ with a pH of 7.4. Several experiments were conducted to manipulate the extracellular environment by eliminating extracellular Ca^2+^, substituting NaCl and KCl with 125 mM KCl, substituting NaCl with 250 mM sucrose, or adding 10% *w*/*v* PEG 8000 [15].

### 3.2. Blood Collection and Experimental Design

Ethical approval was obtained from the Ethics Committee of King Saud University Medical City (E-23-7764). Blood samples were obtained from 27 healthy individuals (18 males and 9 females) aged 26–42 years with a BMI of <25, normal CBC results, and no history of chronic disease. All participants signed informed consent according to the Declaration of Helsinki. Heparinized blood samples were collected to isolate RBCs (2500 RPM, 20 min, RT), and, following washing two times in PBS, cells were stored in PBS or Ca^2+^-free Ringer solution at 4 °C for a maximum of 48 h. Exposure to 10–800 μM of RA was carried out at 5% hematocrit (11.0 × 10^5^ cells/μL) in PBS or Ringer solutions for 24 h at 37 °C. In some experiments, cells were cotreated with 400 μM of RA in the presence or absence of 1 μM of PKC inhibitor staurosporin (STSP), 100 μM of p38 inhibitor SB203580, 20 μM of CK1a inhibitor D4476, or 500 μM of ATP. Additionally, whole blood in EDTA was diluted 1:2 with PBS and exposed to 800 μM RA for 24 h at 37 °C. Control and experimental cells from the same subject were used in distinct experiments to account for potential individual variation [16]. 

### 3.3. Hemolysis and Hemolytic Markers

Supernatants of control and experimental cells were harvested by centrifugation (13,000× *g*, 1 min, RT) to measure hemoglobin (Hb) at 405 nm using a LMPR-A14 microplate reader (Labtron Equipment Ltd., Surrey, UK). The percentage of hemolysis was derived relative to cells suspended in distilled water [17]. AST and LDH activities were detected in the supernatants by the BS-240Pro clinical chemistry analyzer (Mindray Medical International Limited, Shenzhen, China).

### 3.4. Acetylcholine Esterase Activity (AChE)

The activity of AChE in control and experimental hemolysates was photometrically measured using Solarbio’s AChE Activity Assay Kit based on Ellman’s method. Briefly, AChE hydrolyzes acetylthiocholine into thiocholine, which reacts with 5,5-dithiobis-2-nitrobenzoic acid (DTNB), yielding a yellow product (5-mercapto-2-nitrobenzoic acid and dissociated forms) at pH 8.0 (λ_max_ = 412 nm). One unit of enzyme activity is defined as the amount required to produce 1 nM of 5-mercapto-2-nitrobenzoic acid per min per ml of hemolysate.

### 3.5. Eryptosis 

Control and experimental cells were labeled with 1% annexin-V-FITC for 10 min at RT in the dark and analyzed using a Northern Lights^TM^ flow cytometer (Cytek Biosciences, Fremont, CA, USA). FITC was excited by the blue laser at 488 nm, and the emitted green light was captured at 512 nm for a total of 10,000 events. Forward scatter (FSC) and side scatter (SSC) were used as indicators of cell volume and complexity, respectively [18]. 

### 3.6. Intracellular Ca^2+^

Control and experimental cells were stained with 5 μM Fluo4/AM (Ex/Em = 488/520 nm) for 30 min at RT away from light, and 10,000 events were analyzed by flow cytometry [19].

### 3.7. Oxidative Stress

Control and experimental cells were incubated with 10 μM 2′,7′-dichlorodihydrofluorescein diacetate (H_2_DCFDA) for 30 min at 37 °C away from light, and 10,000 events were subsequently analyzed (Ex/Em = 488/520 nm) by flow cytometry [20].

### 3.8. Electrolytes and pH

Control and experimental supernatants were assayed for K^+^, Cl^−^, Na^+^, Mg^+^, and pH using an EXIAS e|1 electrolyte analyzer (EXIAS Medical GmbH, Graz, Austria) based on an ion-selective electrode [21].

### 3.9. Intracellular B_12_

Control and experimental hemolysates were assayed for B_12_ content using Mindray’s CL-1200i chemiluminescence analyzer. In this competitive binding immunoenzymatic assay, B_12_ in the sample competes with paramagnetic microparticles coated with biotinylated B_12_ for binding to alkaline phosphatase-labeled intrinsic factor. Microparticles are then magnetically captured, while other unbound substances are removed by washing. The chemiluminescent signal generated upon addition of a substrate is inversely proportional to the concentration of B_12_ in the sample, which is determined from a calibration curve.

### 3.10. Cellular Morphology

Control and experimental (800 μM) cells were stained with Giemsa stain as per standard protocols. For SEM analysis, cells fixed in 2.5% glutaraldehyde were stained with 1% osmium tetraoxide, dried in 50–100% ethanol, and visualized using the JSM-7610F ultra-high-resolution Schottky field emission scanning electron microscope at 15.0 kV (JEOL Co., Ltd., Akishima, Tokyo, Japan) [15].

### 3.11. Systemic Toxicity

A CBC of control and experimental whole blood samples was performed using Mindray’s BC-6200 hematology analyzer [22].

### 3.12. Erythrocyte Sedimentation Rate (ESR)

The rate of vertical sedimentation (mm/h) of control and experimental RBCs in whole blood was recorded using Westergren tubes [23].

### 3.13. Statistics

Results are presented as means ± SD of three independent experiments. Flow cytometric data were analyzed by FlowJo^TM^ v10.7.2 (Becton, Dickinson, and Company, Ashland, OR, USA), and all statistical analyses were performed by GraphPad Prism v9.2.0 (GraphPad Software, Inc., San Diego, CA, USA). Two experimental groups were analyzed by the unpaired, two-tailed Student’s *t*-test, while three or more groups were analyzed by a one-way ANOVA with Dunnett’s correction. Statistical significance was defined by a *p* value of <0.05.

## 4. Discussion

CAA remains a challenging side effect of current chemotherapy, and investigating the toxicity of potential anticancer therapeutics to erythrocytes is therefore of utmost importance. In this work, we have shown for the first time that anticancer concentrations of RA (10–800 μM) stimulate hemolysis and eryptosis characterized by AST and LDH leakage, PS translocation, dysregulated ion trafficking, Ca^2+^ accumulation, cell shrinkage, and B_12_ entrapment. Both hemolysis and eryptosis were Ca^2+^-dependent and were sensitive to isosmotic sucrose. The toxicity of RA was blunted by energy replenishment and PEG 8000 and was mediated through p38 MAPK, CK1α, and PKC.

In contrast to a previous report showing that RA is not toxic to PBMCs [8], our findings indicate that RA exhibits a dual effect on RBCs (Figure 1), being hemolytic at 200–800 μM and antihemolytic at 50 μM under hypotonic conditions. In addition to its oxidative potential, circulating naked Hb undergoes glomerular filtration and precipitates in kidney tubules, giving rise to renal failure [24]. Prevention of hypotonic lysis, on the other hand, suggests that RA intercalates into the lipid bilayer and expands the membrane, allowing the cell to withstand more water influx relative to untreated cells before hemolysis ensues. Counteracting hemolysis ostensibly by increasing cellular volume has previously been observed with *Ginkgo biloba* leaf extract [25] and mangostin [15].

The central finding in this study is the pro-eryptotic activity of RA (Figure 2). Translocation of PS to the outer membrane leaflet is observed in senescent, infected, and damaged cells. It serves as a binding site for phagocyte receptors to identify and engulf these cells to prevent their extended presence in the circulation, which could lead to intravascular hemolysis. Although suicidal RBC death is an effective defense mechanism that ensures the elimination of dysfunctional cells, premature eryptosis, as elicited by RA in this study, results in excessive removal of circulating cells, which outweighs the rate of erythropoiesis in the bone marrow, culminating in anemia [26]. Importantly, the morphological alterations seen in eryptotic cells and elevated ESR (Figure 5) compromise their deformability, impede blood flow, and increase the risk of thromboembolic events [27]. Also, patients with excessive eryptosis are prone to vaso-occlusive lesions because eryptotic cells adhere to the endothelial wall through CXCL16/SR-PSOX [28].

Of note, RA-induced eryptosis was found to be sensitive to the availability of isosmotic sucrose in the medium (Figure 2). Although the exact mechanism through which sucrose acts as an anti-eryptotic agent still eludes us, we propose the following three scenarios: First, sucrose may block Cl^–^ efflux (Figure 4) and thus reduce fluid loss and cell shrinkage. Second, sucrose may prevent water influx through colloid osmotic pressure because it is a non-penetrating solute. Third, sucrose, being rich in hydrogen-accepting sites, may bind RA and reduce its activity [29]. Further elucidation of these possible scenarios is warranted for future studies.

We have also found that RA elevates intracellular Ca^2+^ (Figure 3), which is a characteristic sign of eryptotic cells. Ca^2+^ regulates the activity of numerous enzymes that preserve the integrity of the cell membrane. Scramblases, flippases, and floppases maintain the orientation of phospholipids within the plasma membrane and are subject to regulation by Ca^2+^ ions. Excessive buildup of Ca^2+^ inside the cell thus leads to dysfunctional enzymatic activity, resulting in loss of membrane asymmetry and PS externalization (Figure 2). The physiological relevance of Ca^2+^ extends to other functions, including signal transduction, motility, and transcriptional regulation [30]. Interestingly, our data reveal that Ca^2+^ are essential to the full hemolytic and eryptotic activities of RA (Figure 3), which indicates that Ca^2+^ acts upstream of PS externalization and membrane rupture. However, since the nominal absence of Ca^2+^ failed to fully abrogate the toxicity of RA, the involvement of other necessary mechanisms is strongly suggested.

Hyperactivity of Ca^2+^-sensitive K^+^ channels secondary to Ca^2+^ buildup leads to KCl efflux, followed by water exit, leading to severe loss of cellular volume and eventual cell shrinkage (Figure 4). Blocking KCl exit by increasing extracellular KCl to 125 mM aggravated the hemolytic effect of RA but failed to protect against its eryptotic effect. This observation suggests that preventing KCl loss either renders existing mechanisms more detrimental to membrane integrity or allows other additional mechanisms to be targeted. In the case of eryptosis, the result indicates that KCl efflux is not necessary for RA-induced PS exposure and Ca^2+^ accumulation, thus activating other essential mechanisms. 

Very few studies have examined the acidity of the extracellular milieu of erythrocytes subjected to chemical stress. In this work, we observed decreased extracellular pH in RA-treated cells (Figure 6), ostensibly due to the buildup of lactic acid from excessive glycolysis. Along those lines, our results suggest that energy replenishment rescues the cells from RA toxicity (Figure 7), an observation pointing at energy turnover being a target of RA in erythrocytes. It is worth mentioning that lactic acid accumulation has been observed in RBCs following exposure to methylglyoxal [31] and tashinone IIA [32], two other eryptotic compounds.

Erythroblasts require B_12_ during their growth and differentiation for DNA synthesis, and a lack of it leads to halted erythropoiesis and the generation of macroerythrocytes characteristic of megaloblastic anemia. Exposure to RA caused significant entrapment of B_12_ inside the cells (Figure 6), suggesting that RA interferes with cobalamin transport and trafficking. Earlier studies have demonstrated that B_12_ uptake occurs in RBCs, and the process seems to be Ca^2+^- or Mg^2+^-dependent [33]. It is plausible, then, to assume that the loss of ionic regulation, most importantly that of Ca^2+^ (Figure 3), is related to the observed intracellular accumulation of B_12_ (Figure 6). Further examination of this association is indeed warranted.

Signal transduction mediators are pivotal for the regulation of erythrocyte survival. Our results show that RA toxicity is significantly blunted in the presence of SB203580 (Figure 7), indicating the participation of p38 MAPK in membrane rupture. Similar to its role in nucleated cells, p38 in RBCs is activated by physical and chemical stress such as hyperosmotic shock [34] and tobacco extract [35]. Notably, pharmacological inhibition of p38 activity also blocked Ca^2+^ accumulation secondary to hyperosmotic shock [34], suggesting that p38 acts upstream of Ca^2+^ signaling. Likewise, co-treatment of cells with RA and STSP (Figure 7) resulted in significantly less hemolysis than that observed in cells treated with RA alone. Previous reports have unequivocally demonstrated that PKC mediates RBC death induced by excessive Ca^2+^ entry [36] and by eryptosis inducers costunolide [37] and temsirolimus [38], among others. Collectively, these studies indicate that PKC acts downstream of Ca^2+^ accumulation but upstream of reactive oxygen species generation. However, since no appreciable oxidative stress was observed in RA-treated cells (Figure 6), PKC activation by RA more likely targets other mechanisms independent of oxidative damage.

Erythrocytes also express CK1α, which plays a significant role in cell differentiation, apoptosis, survival, and stress response [39]. Zelenak et al. reported that CK1α mediates erythrocyte death upon energy deprivation and that D4476 reverses Ca^2+^ accumulation and subsequent cell shrinkage [40], indicating that, like p38, CK1α operates upstream of Ca^2+^ signaling. Since RA also exhausts the intracellular ATP pool (Figure 6), it is speculated that RA activates a CK1α/Ca^2+^/ATP molecular axis that eventually culminates in cell death. Congruently, we have recently reported that both CK1α and metabolic exhaustion, but not Ca^2+^ signaling, are responsible for the toxicity of deguelin in RBCs [22].

Unlike sucrose, urea is a penetrating solute, and 300 mOsm in the presence of isotonic NaCl thus ensures equilibrium is reached inside and outside the cells. Other mechanisms suspected to mediate the protective effects of urea include inhibiting sphingomyelinase activity and modulating the Na^+^-K^+^-ATPase pump, KCl cotransport, or Na^+^-K^+^-2Cl^−^ cotransport [41]. However, among all identified RA inhibitors, PEG thoroughly abrogated the toxic effects of RA, which strongly suggests physical partitioning of either corpuscles or RA molecules, which is supported by its antihemolytic properties demonstrated against shear stress [42]. It is also presumed that membrane pores formed by RA are smaller in size than the hydrodynamic radius of PEG. Future studies should probe whether RA-based PEGylated nanomedicines and pharmaceuticals retain their anticancer activities with lower hemolytic potential.

Our studies on whole blood have discerned that RA toxicity extends to reticulocytes, leukocytes, and platelets. Even in the presence of plasma proteins, mature RBCs were susceptible to RA, which also depleted Hb stores (Figure 8) and reduced corpuscular size (Figure 4). This indicates a lack of inhibitory effects of complement proteins on RA, unlike the susceptibility of sanguinarine [43]. Alarmingly, RA was also cytotoxic to reticulocytes (Figure 9), possibly suggesting that it may antagonize the bone marrow response to a falling RBC count. Such implications of systemic RA toxicity require further examination in vivo.

The immunomodulatory effects of RA have been described in the literature. In particular, RA has been shown to induce apoptosis in T cells in an experimental arthritis model and to reduce neutrophils and eosinophils in the nasal lavage fluid of seasonal allergic rhinoconjunctivitis patients [44]. In line with these observations, our data show that RA adversely affects the viability of neutrophils, lymphocytes, and monocytes in whole blood (Figure 10). Furthermore, RA exhibits antiplatelet activity with the formation of enlarged cells (Figure 11), consistent with previous reports showing that RA inhibits platelet adhesion [45] and aggregation [46]. Functional and mechanistic studies on the effects of RA on leukocytes and platelets are indeed warranted.

In conclusion, this work reveals that anticancer concentrations of RA trigger hemolysis and eryptosis in human erythrocytes. RA-induced RBC death was found to be Ca^2+^-dependent and sucrose-sensitive and was characterized by membrane perforation, breakdown of phospholipid asymmetry with PS translocation, Ca^2+^ accumulation, cell shrinkage, dysregulated ion transport and B_12_ trafficking, and acanthocyte formation. These toxic manifestations were mediated through energy deprivation, p38 MAPK, PKC, and CK1α, and could be reversed by isosmotic urea and PEG. Altogether, our findings argue for a cautious consideration of RA in anticancer therapy and the utilization of antihemolytic and antieryptotic agents as possible adjuvants.

## Data Availability

Data are contained within the article.
